# Comprehensive analysis on diagnostic value of circulating miRNAs for patients with ovarian cancer

**DOI:** 10.18632/oncotarget.18129

**Published:** 2017-05-24

**Authors:** Huiqing Wang, Tingting Wang, Wenpei Shi, Yuan Liu, Lizhang Chen, Zhanzhan Li

**Affiliations:** ^1^ Department of Social Medicine and Health Management, Xiangya School of Public Health, Central South University, Changsha, Hunan Province 410078, China; ^2^ Department of Oncology, Xiangya Hospital, Central South University, Changsha, Hunan Province 410008, China

**Keywords:** diagnostic, ovarian cancer, miRNA, meta-analysis

## Abstract

We performed a meta-analysis to assess the diagnostic accuracy of circulating miRNA for patients with ovarian cancer. We systematically searched several online databases, including PubMed, Web of Science, Chinese National Knowledge Infrastructure, and Wanfang from inception to February 20, 2017. We used the bivariate mixed-effect models to pool positive likelihood ratios, negative likelihood ratios, diagnostic odds ratios and their 95% CI confidence intervals (CIs). We used the Quality Assessment of Diagnostic Accuracy Studies 2 for quality assessment of diagnostic accuracy studies. This meta-analysis included ten studies with the number of 1356 participants. The pooled sensitivity and specificity were 0.75 (95%CI: 0.69-0.80) and 0.75 (95%CI: 0.69-0.81). We also calculated the positive likelihood ratios (3.03, 95%CI: 2.44-3.76), and negative likelihood ratios (0.33, 95%CI: 0.27-0.41). The diagnostic odds ratio was 9.09 (95%CI: 6.51-12.69). The summary receiver operator characteristic was 0.82 (95%CI: 0.78-0.85). Sensitivity analysis showed similar results. No publication bias existed (t=0.380, *P*=0.712). The diagnostic ability of miRNAs were moderate for ovarian cancer. Further research was required to obtain accurate results.

## INTRODUCTION

Ovarian cancer is one of the most common malignant tumor of female reproductive organs, its incidence is the third, and lowers than that of cervical cancer and endometrial cancer. However, the fatality is highest among three kinds of tumors. Ovarian cancer threatened the women’ life and health seriously [1]. Currently, 70-80% of patients had been in advanced stage when they was diagnosed because the specific clinical manifestation and early diagnostic methods were scare, usually followed by abdominal and pelvic metastasis. The five-year survival rates of patients with advanced ovarian cancer were only 20-30% compared to over 90% for those with early stage ovarian cancer [2]. Many diagnosis index had been applied in the clinical practice such as carbohydrate antigen 125 (CA125), CA199, carcinoembryonic antigen (CEA), and human epididymis protein 4 (HE4). CA125 was the most widely used in clinical diagnostic, and both of its sensitivity and specificity ranged from 70% to 80% [3, 4]. Sometimes false positive results could appear in some cancer types such as endometriosis, adenomyosis, pelvic inflammation, hysteromyoma and benign ovarian cysts [5]. New biomarkers with high diagnostic value were urgently needed for ovarian cancer with the aim of early diagnostic, treatment and improving survival rate and quality of life.

In recent years, differential expression of circulating miRNA was observed in different types of tumor or cancer. The development of new technology in detecting RNA from tiny amount of the cells thus obtained. It was quite convenient for detecting miRNA from serum and plasma, which made miRNA become a new diagnostic biomarker [6, 7]. Many studies had reported the diagnostic values of circulating miRNA for ovarian cancer, but the results remained inconsistent because of some potential factors such as sample sizes, histological types of tumor, specimen sources, examining methods [8, 9]. To estimate the diagnostic values of miRNA for early ovarian cancer, we performed the quantitative analysis through systematical search and following strict criterions of inclusion and exclusion.

## RESULTS

### Study selection

**Figure 1:**
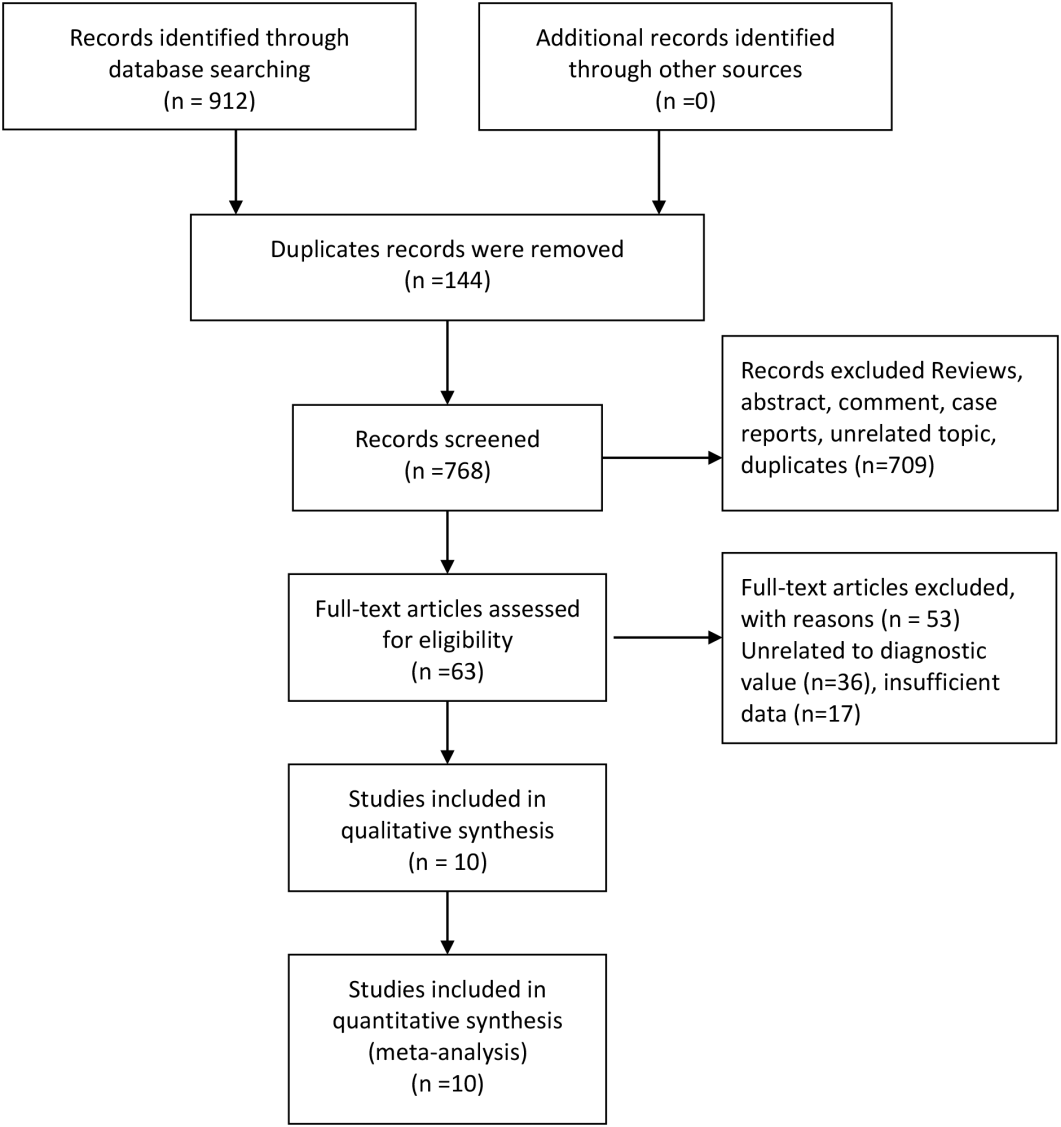
Flow diagram of studies selection process

Figure 1 presented the process of study screening. Our initial search returned 912 records, and no additional records identified through other sources. After excluding duplicates and scanning the titles and abstracts, we got 63 full-text articles for eligibility. After reviewing the full-text, we put 10 records (12 studies) in the final qualitative and quantitative synthesis [10–19].

### Study characteristics and quality assessment

The general characteristics were presented in Table 1. These articles were collected from 2012 to 2016. The number of participants in these studies were 1356. The sample size ranged from 30 to 250. Six studies were from China, and the rest of studies were from German, Australia, Korea, and USA, respectively. Two specimens were from plasma, and ten ones were from serum. According to the QUADS-2 scale, the mean score of all studies were 9.2 scores. The quality of included studies were high.

**Table 1:** General characteristic of included studies in the meta-analysis

First author	Year	Country	Age range(y)	Sample source	Sample size	TP	FP	FN	TN	QUADS
Kuhlmann	2014	Germany	18-81	Serum	98	33	3	30	32	9
Kan	2012	Australia	30-79	Serum	56	22	15	6	13	8
Hong	2013	China	18-70	Serum	131	85	5	11	30	9
Guo	2013	China	-	Serum	100	40	12	10	38	10
Zeng1	2016	China	19-72	Serum	122	27	18	13	64	8
Zeng2	2016	China	19-72	Serum	122	30	24	12	58	8
Gao1	2015	China	>18	Serum	143	67	15	26	35	9
Gao2	2015	China	>18	Serum	143	64	14	29	36	9
Liang	2015	China	-	Serum	119	63	32	21	103	10
Chung	2013	Korea	42-71	Serum	30	14	1	4	11	10
Suryawanshi	2013	USA	-	Plasma	42	19	9	2	11	9
Zheng	2013	China	56.5/53.7	Plasma	250	107	18	43	82	11

### Pooled diagnostic values

**Figure 2:**
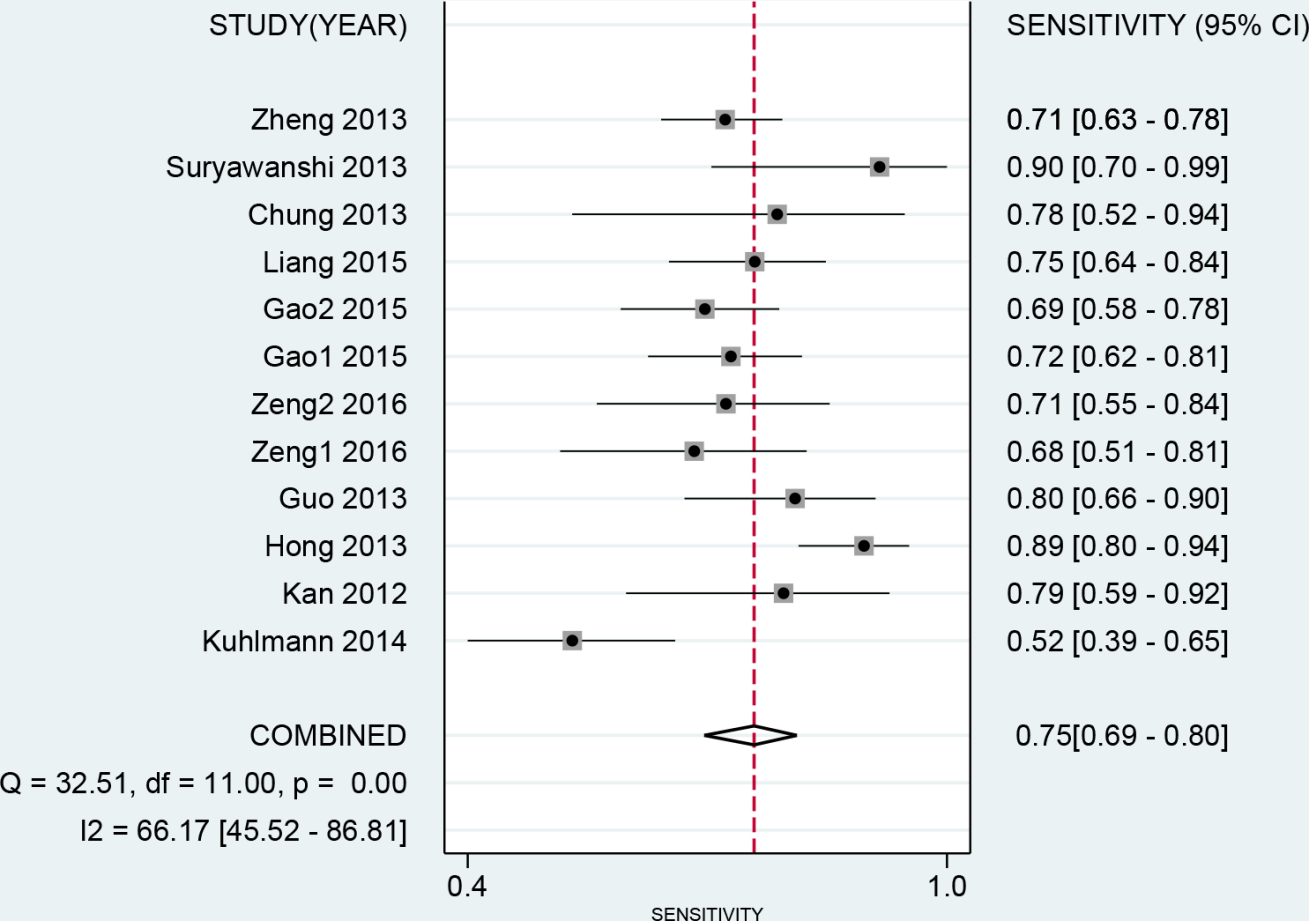
Forest plot of pooled and each study’s sensitivity of miRNAs for ovarian cancer

**Figure 3:**
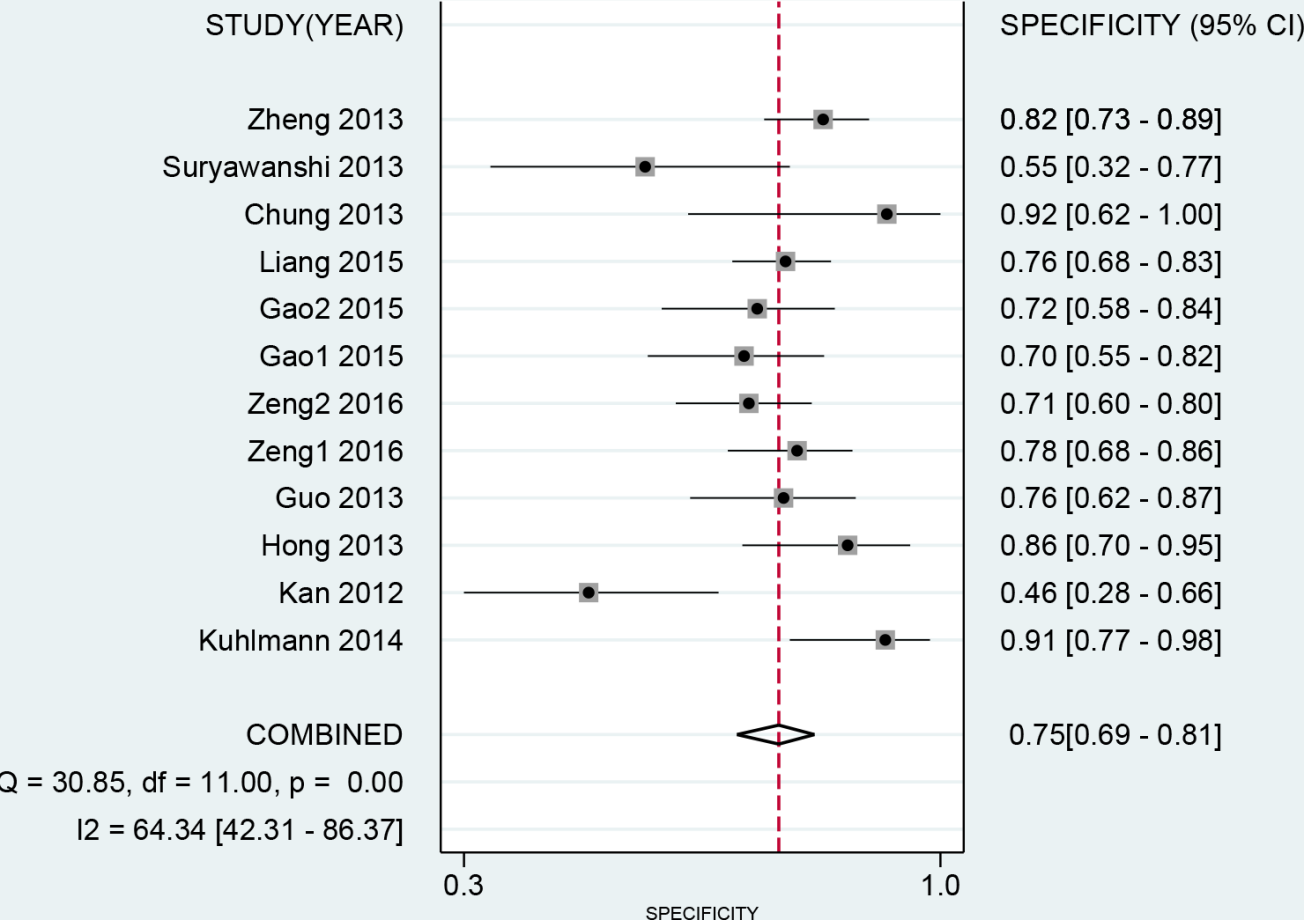
Forest plot of pooled and each study’s specificity of miRNAs for ovarian cancer

**Figure 4:**
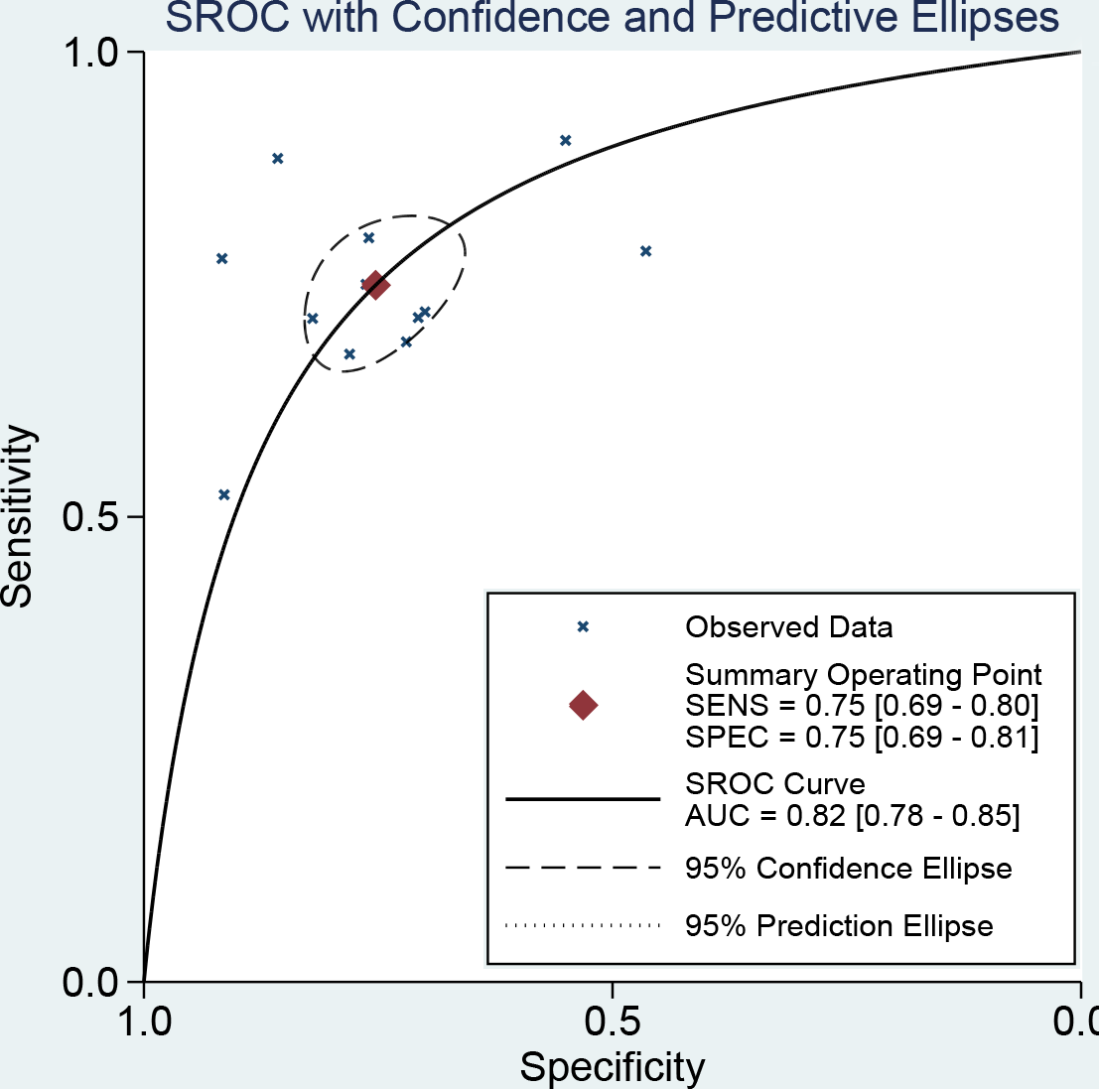
The symmetric receiver operating characteristic curve of miRNAs for ovarian cancer

**Figure 5:**
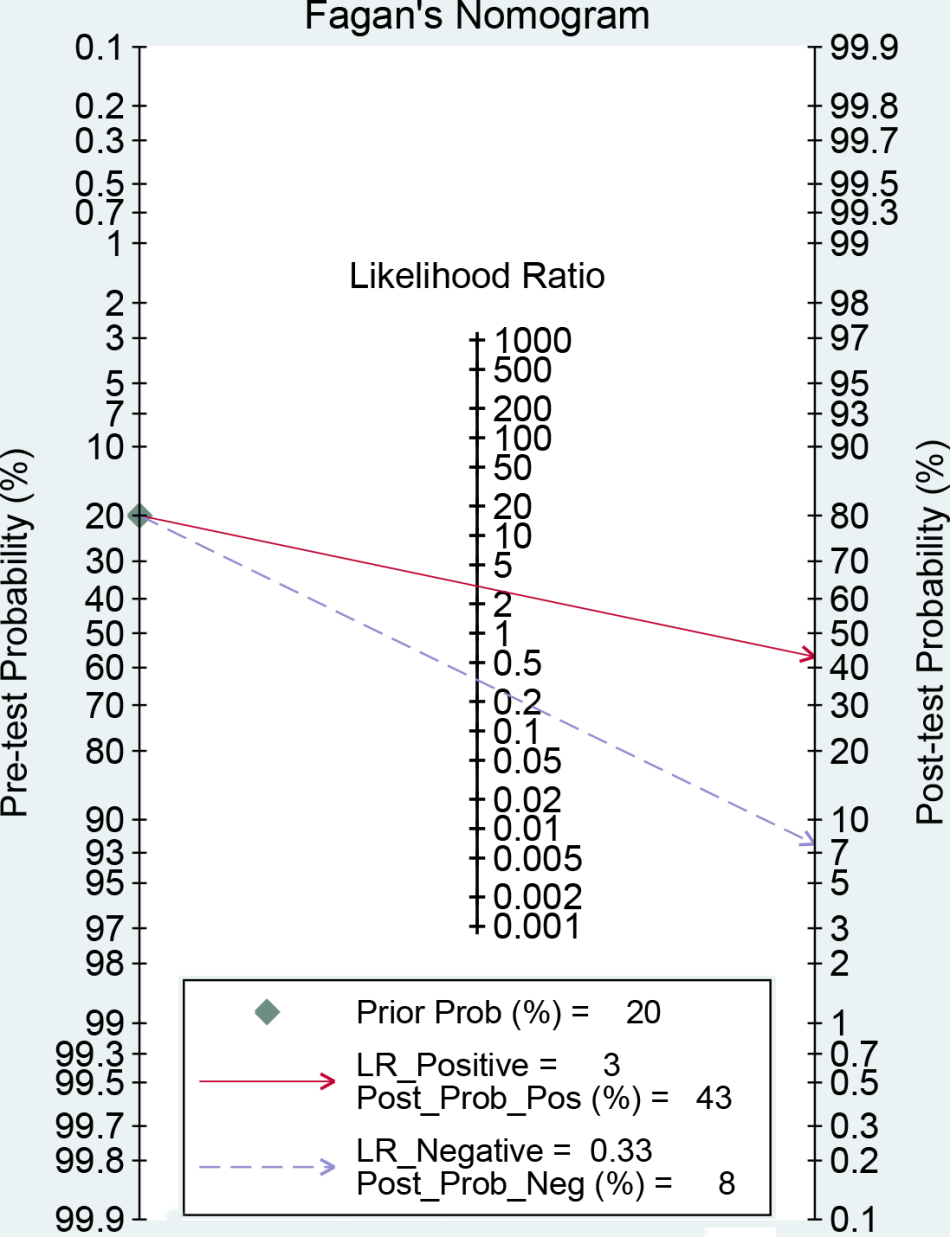
Fagan diagram evaluating the overall diagnostic value of miRNAs for ovarian cancer (if the pre-test probability was 20% according to the circulating miRNA, then post-probability was almost 40% according the calculated positive likelihood ratio)

The results of threshold test showed no association between sensitivity and specificity (r=0.091, *P*=0.790). This result allowed us to perform the analysis through the bivariate mixed-effect models. We used the random-effect models to pool the estimations. Twelve studies were included for sensitivity and specificity. The pooled sensitivity and specificity were 0.75 (95%CI: 0.69-0.80, I^2^=66.17%, Figure 2) and 0.75 (95%CI: 0.69-0.81, I^2^=64.34%, Figure 3). We also calculated the positive likelihood ratios (PLRs), negative likelihood ratios (NLRs), and the results shows the diagnostic ability of miRNA were relatively high (PLR: 3.03, 95%CI: 2.44-3.76; NLR: 0.33, 95%CI: 0.27-0.41). The diagnostic odds ratio was 9.09 (95%CI: 6.51-12.69). The summary receiver operator characteristic is 0.82 (95%CI: 0.78-0.85, Figure 4). We used the Fagan to assess the clinical application. The results showed the post-test probability about 43% with 20% of pre-test probability. The diagnostic ability were moderate (Figure 5).

### Sensitivity analysis and publication bias

**Figure 6:**
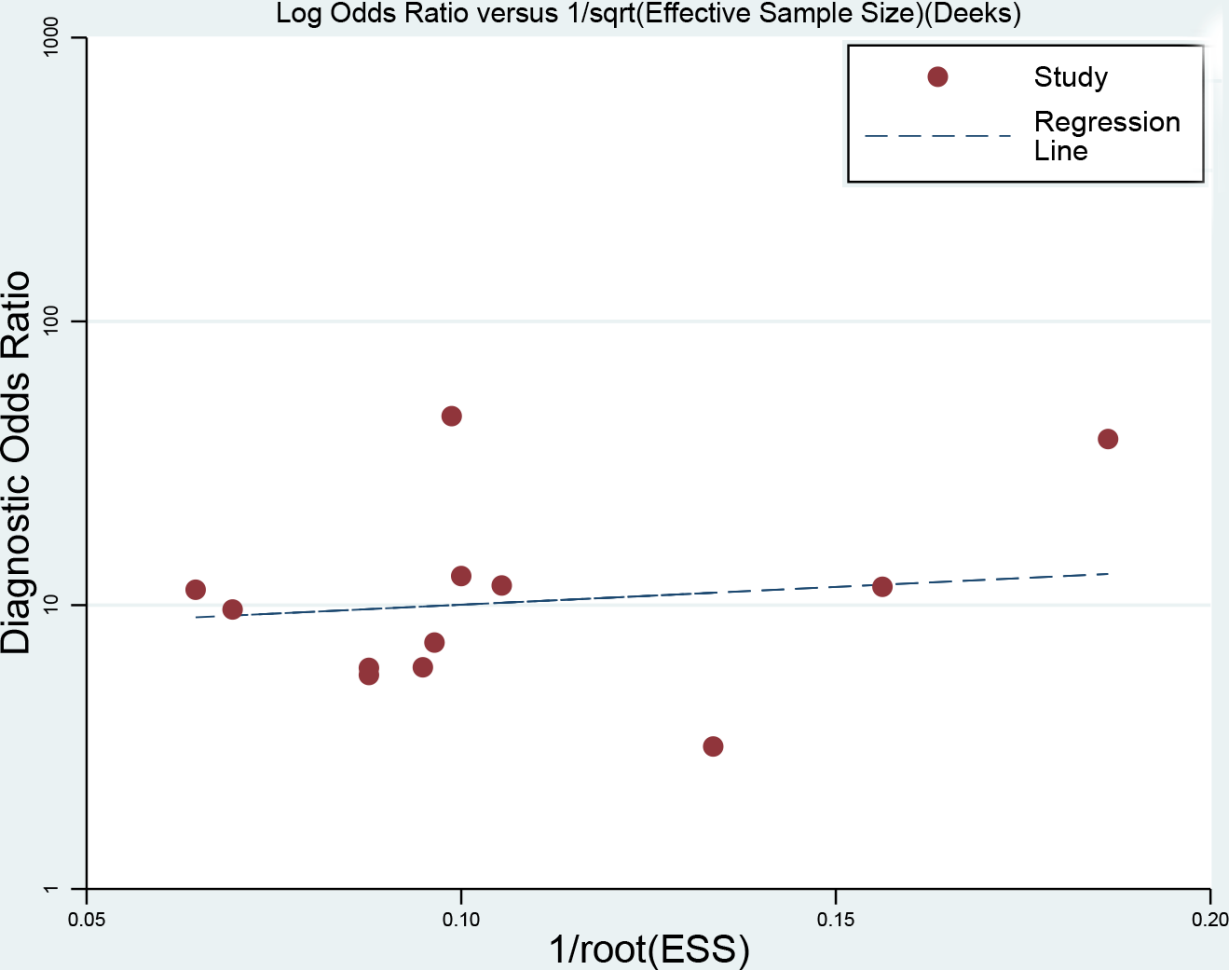
Deek’s funnel plot to evaluate the publication bias (angle between regression line and X-axis comes closer to 0°, smaller possibility of publication bias)

To explore the potential sources of heterogeneity and stability of results across the studies, we carried a sensitivity analysis by excluding two studies conducted in plasma. The pooled sensitivity and specificity were 0.74 (95%CI: 0.67-0.80) and 0.76 (95%CI: 0.69-0.81,). The results shows the diagnostic ability of miRNA were relatively stable (PLR: 3.05, 95%CI: 2.38-3.91; NLR: 0.33, 95%CI: 0.27-0.44). The diagnostic odds ratio was 8.84 (95%CI: 5.87-13.33). The summary receiver operator characteristic was 0.81(95%CI: 0.78-0.85). The whole results kept stable. We used Deek’s plot to test the publication bias. The bias test shown there was no existence of publication bias (t=0.380, *P*=0.712, Figure 6).

## DISCUSSION

The present study found that circulating miRNA could be a moderate diagnostic biomarker for ovarian cancer. The sensitivity and specificity of diagnostic were 75% and 75% with AUC of 0.82. This diagnostic ability was almost equal to CA125, the most widely used serum index, with sensitivity of 0.74, specificity of 0.83, and AUC of 0.85 [20]. There were still 20%-30% of patients with ovarian cancer that can’t be confirmed. More accurate diagnostic biomarkers were in great need. The miRNA was a noncoding single-stranded RNA encoded by endogenous gene, with the length of 21-24 nucleotides. miRNAs was associated with the process of cell differentiation, biological development, and disease progression because their involvement in expression and regulation of post-transcriptional gene. Mitchell segregated 125 RNA with 18-24 nucleotides from health population, and built a RNA pool. The sequencing analysis showed 72.8% of RNAs were known and only 3.2% were unknown, which indicated that mature RNAs can be detected in human plasma [21]. He further found the expression of miR-15b, miR-16, and miR-24 in human serum and plasma. He also approved that these endogenic miRNAs can be stably stored in different temperature, repetitive freeze-thawing, and effects of extrinsic miRNA, and confirmed that miRNA can be stable and freed from being degraded by endogenic RNase in human circulation blood [21, 22]. The miRNA characterized by stability, detectability, and specificity of tumor tissues makes it possible to become a noninvasive biomarker in the clinical diagnostic of tumor. Though the literature and the Gene Expression Omnibus showed some profiling data of the circulating microRNAs for ovarian cancer, the circulating microRNAs data for ovarian cancer were not currently sufficient to analyze their diagnostic potential. More analyses are needed.

Our results found that both of the sensitivity and specificity of miRNA were 0.75, and the misdiagnosis rate was 0.25, higher than 0.15, which indicated miRNA was not high specificity diagnostic index. The combined NLR and PLR were 0.33 and 3.03. According to the criterion of high diagnostic value (PLR>5, NLR<0.2), the present results showed that miRNA was weak for ovarian diagnostic. However, the AUC fallen into 0.7 to 0.9. This means the diagnostic value of miRNA was moderate.

The threshold effect was an important factor of heterogeneity for screening test. The reasons could be that screening test cannot meet the requirement of randomized controlled trials, and different study had different conditions. Our result did find there were threshold effects within study. But the heterogeneity among studies were high. This may be related to expression level of overall miRNA. The sensitivity, specificity and DORs results suggested that the heterogeneity were caused by no threshold effects. Having considered the ethnic, sample source, sample size, we did not find other sources, either. We further conducted sensitivity analyses through excluding two study [17, 18]. The heterogeneity did not reduce significantly. We assumed that this situation may be related to examining methods and stage, or could be associated with distribution of circulating miRNA. It just began that miRNA was treated as a diagnostic biomarker for ovarian cancer. The number of relevant study was limited. Therefore, we cannot conduct further subgroup analyses. Most of research data were among Chinese population based on the present search results. The ethnicity differences should be taken into consideration in the future study.

Sum it up, circulating miRNA, as less invasive, simple and operational technique, had possibility of missed diagnosis. But it still have a moderate diagnostic ability, which can improve diagnostic accuracy when combined with CA125 or other biomarkers. It was important to note that large-scale multi-center clinical research were required to obtain more accurate estimations. The future study should pay attention on combined diagnosis from many different kinds of specific miRNAs expression.

## MATERIALS AND METHODS

The ethical approval was not applicable for the present study because this was a study based on published articles. This meta-analysis was conducted in accordance with PRISM (Preferred Reporting Items for Systematic Reviews and Meta-Analyses) Statement [23].

### Literature search

We systematically searched the PubMed, Web of Science, Chinese National Knowledge Infrastructure, and WanFang, from inception to February 20, 2017. We used the following search subject heading and keywords to identify the relevant articles about diagnostic values of microRNA for ovarian carcinoma: microRNA or micro-RNA, miRNA or mi-RNA, ovarian cancer, ovarian carcinoma, ovarian tumor, diagnosis or diagnostic value, sensitivity, specificity, receiver operating characteristics curve. The relevant lists of articles and reviews were also retrieved to obtain eligible studies. The search language was restricted in Chinese and English.

### Selection criteria

Two authors (Y.L and Z.L) independently performed the searches according to a set of standards. The third author solved any disagreements. The included study had to meet the following criteria: I) Study about the diagnostic value of miRNA for ovarian carcinoma with available full text. II) All cases were confirmed by gold standard (criteria recommended by International Federation of Gynecology and Obstetrics). III) The miRNA was located in plasma or serum. IV) Sufficient data was provided for further pooling, including true positive (TP), false positive (FP), false negative (FN), and true negative (TN). Duplicates, study with incomplete data, reviews, cases report, and comment were excluded. The latest data was used for duplicates.

### Data extraction

Two authors (T.W. and W.S.) performed the data extraction. We used a standardized Excel sheet for extracting the following information from each include study: the first author, year of publication, country, range of age (mean), sample source, sample size, and four values for analyses (TP, FP, TN, FN).

### Quality assessment

According to the Cochrane Handbook for Systematic Reviews, we used the Quality Assessment of Diagnostic Accuracy Studies 2 (QUADAS-2) for quality assessment of diagnostic accuracy studies [24]. This assessment tool includes four main items, and each item includes several sub-items with low, high and unclear risk levels. We treated 1 score as low risk, -1 score for high risk, and zero for unclear risk. Studies with more than 7 scores were considered to be high quality.

### Statistical analysis

We firstly calculated the spearman correlation coefficient between sensitivity and specificity to test the threshold effect. No threshold effect was observed (r=0.091, *P*=0.790) [25]. The bivariate mixed effects models were used to pool the sensitivity, specificity, PLR, NLR, DORs with 95% confidence intervals (CI) [26]. We used the Q test to examine the heterogeneity qualitatively, and I^2^ statistic to assess the heterogeneity quantitatively. *P*<0.05, or I^2^>50% indicated the presence of heterogeneity [27]. We estimated the area under the summary receiver operator characteristic cure with 95%CI (AUC). AUC>0.5 represented a good diagnostic ability (0.90-1.00 =excellent, 0.80-0.90 =good, 0.7-0.8 =fair, 0.6-0.7 =poor, 0.50-0.60 =fail) [28]. We also used the Fagan plots to show the prior probability and posterior test probability, and the publication bias was assessed by Deek’s funnel plot [29]. All statistical analyses were completed on Stata 14.0 (Corp. College Station TX, USA), and *P*<0.05 was considered to be significant.

## Abbreviations

QUADAS-2: quality assessment of diagnostic accuracy studies 2
TP: true positives
FP: false positives
FN: false negatives
TN: true negatives
ROC: receiver operating characteristic
SROC: summary receiver operator characteristic
PLRs: positive likelihood ratios
NLRs: negative likelihood ratios
DORs: diagnostic odds ratios
CI: confidence intervals
CA125: carbohydrate antigen 125
CEA: carcinoembryonic antigen
HE4: human epididymis protein 4
